# Isolated spleen tuberculosis in an immunocompetent patient, a rare case report

**DOI:** 10.1016/j.ijscr.2021.105966

**Published:** 2021-05-12

**Authors:** Diah Asih Lestari, Nur Rahadiani, Ridho Ardhi Syaiful

**Affiliations:** a Digestive Surgery Division, Department of Surgery, Faculty of Medicine, Universitas Indonesia Cipto Mangunkusumo Hospital, Jl Diponegoro No 71, Salemba, Jakarta Pusat 10430, Indonesia; b Department of Anatomical Pathology, Faculty of Medicine, Universitas Indonesia Cipto Mangunkusumo Hospital, Jl Diponegoro No 71, Salemba, Jakarta Pusat 10430, Indonesia

**Keywords:** Case report, Immunocompetent, Infection, Spleen, Tuberculosis

## Abstract

**Introduction:**

Tuberculosis (TB), as a major public health concern, is affecting almost 10 million people globally. At present, diagnostic and screening efforts mainly focus on positive smear results. Therefore, the number of extra pulmonary and negative sputum TB is rising and hampering the diagnosis and treatment process due to the large number of false negatives. Rare cases such as solitary splenic TB are usually seen in patients with splenic abnormalities, spleen trauma, immunosuppression, sickle cell disease, pyogenic infections, etc.

**Presentation of case:**

A 40-year-old female with no comorbidity came with chief complaint of early satiety every mealtime and epigastric pain in the last 6 months prior to admission. There was no significant positive examination except for positive IGRA test and enlargement of spleen with multiple cystic lesions on abdominal CT. We performed laparotomy with splenectomy followed by a histopathology examination which showed features of primary tubercular abscess.

**Discussion:**

In the immunocompromised patient, the visceral abdomen is usually involved and a part of miliary TB. However, this case revealed the rare possibility of a healthy person with primary isolated tubercular splenic abscess while being immunocompetent and lacking any comorbidity.

**Conclusion:**

Splenic TB diagnosis is difficult in patients lacking pulmonary involvement and without specific symptoms. Thorough examinations and clinical expertise are needed to provide accurate diagnosis and treat uncommon forms of TB and cases with negative smear results in consideration of rising prevalence and difficult disease control.

## Highlights

• A case of isolated splenic tuberculosis was found in an immunocompetent patient.
• In this case, TB is again the great imitator, with symptoms similar to malignancy.
• Conventional diagnosis is inconclusive, and only postsurgical pathology confirms TB.
• Anti-TB drugs are first line for splenic TB, with surgery for complicated cases.
• TB is a possible diagnosis for splenic mass, even in immunocompetent patients

## 1 Introduction

Tuberculosis (TB) is a significant health problem in developing countries and is still one of the most common and fatal infectious disease [1]. In 2014, TB affected almost 10 million people worldwide and the current “End TB Strategy” from the World Health Organization, which started in 2016, is now striving towards a world free of TB [2].

Tuberculosis is located primarily in the lung (90%), whereas isolated splenic TB is a rare form of extrapulmonary (EP) TB, common as a secondary involvement in miliary TB [1,3,4]. Risk factors included immunodeficiency, HIV infection, diabetes mellitus, hematologic abnormalities, chronic steroid therapy, and organ transplantation [3,5]. Isolated spleen TB, without other source is very rare in the healthy individual and late diagnosis is common [1,6,7].

Epidemiologically, only few isolated case reports of splenic TB are available from different regions, making the prevalence uncertain [6]. In Indonesia, data from the Ministry of Health showed the incidence of TB was 842,000 case per year in 2018 [8] but there is no clear data about the incidence of EP TB, especially splenic TB.

Splenic TB can be found as a solitary splenic lesion, splenic abscess, or hypersplenism. Splenic abscess is the most dominant case found along showing most symptomatic presentation but often is non-specific in clinical presentation [5,6].

Splenic TB lacks diagnostic criteria [3,9] with most common symptoms including splenomegaly (13.2–100%), fever (82.3%), and weight loss and fatigue (44.12%). Hence, possible misdiagnosis included spleen carcinoma, tumour metastases, hemangioma, lymphoma, or splenic abscess, moreso if the patients denied any history of tuberculosis [1]. Meanwhile, abdominal pain is usually a rare presentation of splenic rupture [10]. Other reported features included leucocytosis and increased erythrocyte sedimentation rate (ESR) [6].

Organ involvement is usually a confirmation for splenic abscess and therefore it is not routine to include invasive diagnostic modalities [9]. Isolated splenic TB diagnosis is confirmed through pathological examination of fine needle aspiration biopsy, splenic biopsy, or splenectomy specimen [3,6,11]. Meanwhile, assessment of the surrounding organs, need for surgical intervention, and therapy response use ultrasonography and CT scan [9]. Ultrasonography is used localize lesions for aspiration and CT scan can reveal splenic abnormalities, possible biopsy, or drainage site, and is useful for follow up [6,10,12].

CT findings of splenic TB are commonly presented as micronodular type (miliary) and (rarely) macronodular type. The micronodular type shows tiny multiple low-attenuating nodules with central enhancement (acute stage) and calcification (chronic stage). Meanwhile, the macronodular type shows diffuse splenomegaly with single or multiple large low-attenuating tumour like mass, calcification, and peripheral enhancement in advanced lesions.

Due to the limitations of spleen CT, some diseases may have similar hypodense multiple splenic lesions such as metastatic cancer, malignant lymphoma, hemangioma, echinococcal cysts, hydatid cyst, or sometimes frequent fever in infectious diseases, rendering common misdiagnosis [1,3,13]. Some CT scan characteristics for splenic TB included saccular foci, solitary/multiple nodules, or hypodense areas in the spleen without calcification [6,14]. Other reported possibilities are pseudotumour appearance and non-pathognomonic round or ovoid nodules [15]. CT scan cannot easily detect typical nodules on the splenic capsule due to their small size [16].

This case report highlighted isolated splenic TB including the clinical presentation, diagnosis, and management in the immunocompetent patient and emphasizes the need for meticulous and prompt diagnosis by clinicians and health workers to address primary visceral TB as an entity [3].

## 2 Case

A 40-year-old female patient came to our surgical outpatient clinic, Cipto Mangunkusumo Hospital Jakarta, with chief complaint of early satiety at mealtime since 6 months prior to admission. The patient also complained of epigastric pain with no other remarkable complaints. Chronic cough, fever, night sweat, anorexia, or weight loss were absent. There was no history of previous TB infection nor prolonged contact with TB patients.

**Fig. 1:**
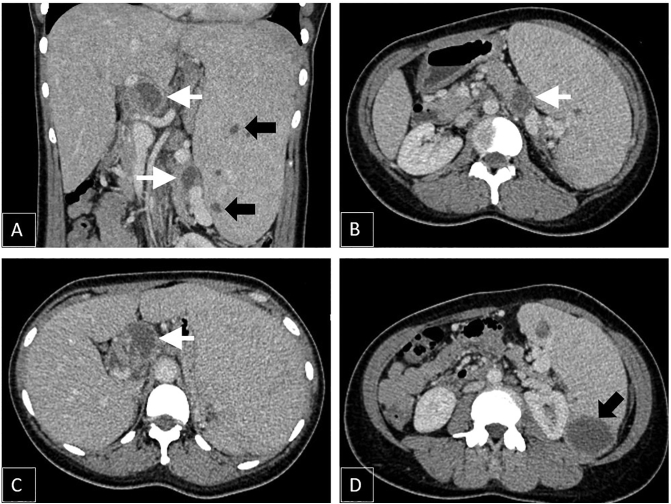
Contrast-enhanced CT (axial and coronal) images A-D showing multiple rim-enhanced cystic lesions within spleen parenchyma (black arrow) with multiple lymph node enlargement with central necrosis (white arrow).

From the physical examination, the prominent finding was isolated enlargement of the patient's spleen (Schuffner IV). Laboratory examination showed leucocytosis and thrombocytosis with positive Interferon Gamma Release Assays (IGRA) test. Anti-Human Immunodeficiency Virus (HIV) test was negative. There were no signs of TB infection from the chest x-ray examination. The CT scan examination revealed spleen enlargement with multiple rim-enhanced cystic lesions mostly within inferior pole parenchyma. There were also cystic lesions within the body and tail of the pancreas and lymph node enlargement of the perihilar and paraaortic region with central necrosis (Fig. 1).

We decided to perform a laparotomy-splenectomy assuming that the splenomegaly and lymphadenopathy were probably caused by malignancy and early satiety was caused by the enlarged spleen pressing the stomach. We gave vaccination pre-operatively to minimize the risk of overwhelming post-splenectomy infection (OPSI).

**Fig. 2:**
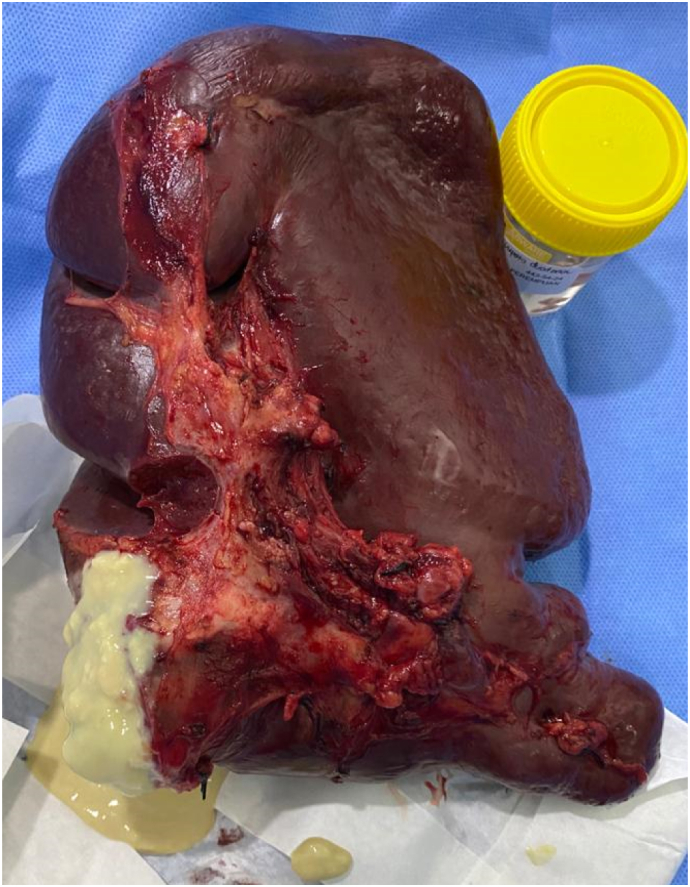
Enlarged spleen with visible caseous necrotic tissue.

Intraoperatively, we found spleen enlargement in the midline which bled easily, adhered to the left lobe of the liver, and pushed the pancreas downward. We also found caseous necrosis tissue at the lateral right of the enlarged spleen that we thought originated from the peri-pancreatic and the splenic hilar lymph nodes (Fig. 2). We performed a splenectomy and lymph node biopsy with minimal difficulties and blood loss. The patient was discharged from the hospital on the 3rd post-operative day with no remarkable complaints.

**Fig. 3:**
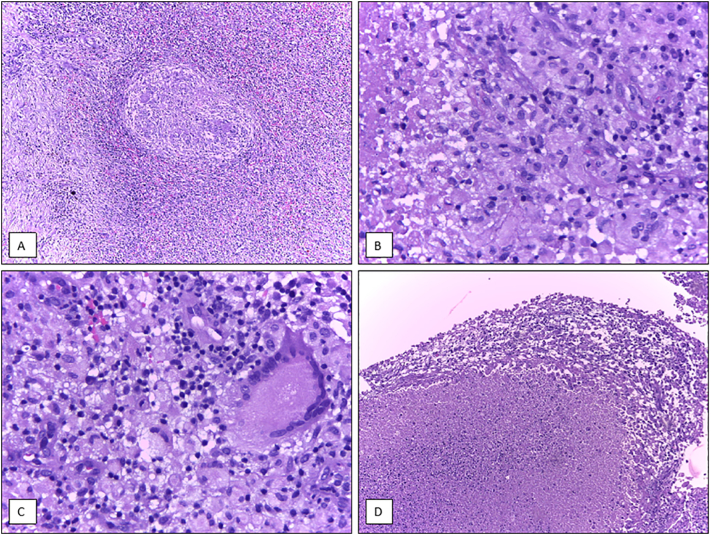
Histopathology examinations of spleen resection showed multiple tubercles (A), consisted of epitheloid cells (B), Langhans multinucleated giant cells (C), and caseous necrosis (D) (Hematoxylin-Eosin, 400×).

The histopathological results showed granulomatous inflammation of the spleen consisted of tubercles, epitheloid cells, and Langhans multinucleated giant cells, along with caseous necrosis (Fig. 3). The morphology was consistent with *Mycobacterium tuberculosis* infection. There was no sequel after the operation and the patient continued with standard regimen for EP TB therapy.

## 3 Discussion

Lacking specific diagnostic criteria, dyspepsia was the only symptom found in our patient as the patient did not even notice a lump in her left upper quadrant abdomen (splenomegaly). The only supporting laboratory data was a positive IGRA test.

In our case, the CT scan showed findings like malignancy. Ultrasound or CT guided percutaneous fine needle aspiration biopsy is a better diagnostic procedure which has been reported to be accurate and safe in diagnosing splenic lesions [1,3,15]. For etiological diagnosis, a histopathological examination is necessary showing lymphocytes, epithelioid granulomas composed of epithelioid cells aggregates, and Langhans' giant cells with various degree of central caseous necrosis in both the white and red pulps [3,4].

Laparotomy or splenectomy may be required in cases with small nodules that might be missed on needle biopsy [3]. Polymerase Chain Reaction (PCR) can be used for further confirmation together with histopathology examination, performing a tubercular culture and antibiotics sensitivity test considering multi drug resistant (MDR) TB surge and extensively drug resistant (XDR) tuberculosis cases. [3,16].

From the reported cases, almost all made first diagnosis by radiology examinations and pathological results. In the case of biopsy failure, diagnosis is made through laparoscopic splenectomy. Unnecessary laparotomy can be avoided by using laparoscopy and is recommended for any form of splenic biopsy [17]. For definitive diagnosis of undefined cases, surgery is still the gold standard [1].

To date, the ideal method to confirm the diagnosis is histopathological examination [1,3]. However, the invasive nature causes reluctance in many patients, thus resulting in delayed or wrong diagnosis [3].

Standard anti-TB regimen should be taken pre-operatively and continued post-operatively if a surgery is carried out [3]. As in other EP cases, the anti-TB therapy shows good response perhaps due to the relative paucity of tissue organisms and excellent tissue penetration [18]. From the few controlled trials on EP TB patient treatment, a 12-month therapy is strongly suggested with prolonged treatment possible if deemed necessary [12].

The pharmacologic response is relatively significant prompting anti-TB drugs as the first line management of splenic TB and splenectomy is rarely required [3,6,19]. However, in the case of abscess formation, inconclusive biopsy specimens, or non-responsive treatment, a surgical treatment is needed [18]. Surgical intervention in primary splenic TB literature is scarce as it was rarely documented, revealing only diagnostic laparoscopic punch biopsies [3,17]. Surgery is also needed in patients with spontaneous rupture of the spleen or failure of anti TB therapy [6,11]. We cannot perform laparoscopic splenectomy in our case because the spleen was already too big posing a difficulty when inserting the trocars, limiting view of surgical field, containing a high risk of bleeding when separating the spleen from adjacent organs, and difficulty when extracting the spleen out of abdominal cavity. This case has been reported in line with the SCARE Guideline [19].

## 4 Conclusions

Immunocompetent patients are rarely found with primary TB splenic abscess and often misdiagnosed. After using multiple approaches and examining possible similar disease entities, we decided to perform laparotomy-splenectomy and lymph node biopsy. Splenic TB was confirmed from the histopathology examination. Therefore, doctors should always keep an open mind and not directly exclude isolated splenic TB diagnosis in immunocompetent patients. We encourage similar case reports to be publicized to increase medical awareness of the rare condition as a disease entity.

## Funding

This research did not receive any specific grant from funding agencies in the public, commercial, or not-for-profit sectors.

## Ethical approval

Ethical approval was exempted by the University of Indonesia and Cipto Mangunkusumo Hospital.

## Consent

Written informed consent was obtained from the patient for publication of this case report and accompanying images. A copy of the written consent is available for review by the Editor-in-Chief of this journal on request

## Provenance and peer review

Not commissioned, externally peer-reviewed.

## CRediT authorship contribution statement

1. Diah Asih Lestari: clinical data collection, manuscript writing and revision, manuscript submission.
2. Nur Rahadiani: histopathological data provision and release, manuscript revision, and manuscript submission approval.
3. Ridho Ardhi Syaiful: surgery operator, case report design, manuscript revision, and manuscript submission approval.

## Declaration of competing interest

None.
